# Off-Resonant Absorption Enhancement in Single Nanowires via Graded Dual-Shell Design

**DOI:** 10.3390/nano10091740

**Published:** 2020-09-02

**Authors:** Wenfu Liu, Xiaolei Guo, Shule Xing, Haizi Yao, Yinling Wang, Liuyang Bai, Qi Wang, Liang Zhang, Dachuan Wu, Yuxiao Zhang, Xiao Wang, Yasha Yi

**Affiliations:** 1 School of Mechanical and Energy Engineering, Huanghuai University, Zhumadian 463000, Henan, China; guoxiaolei@huanghuai.edu.cn (X.G.); xingshule@huanghuai.edu.cn (S.X.); yaohaizi@huanghuai.edu.cn (H.Y.); wangyinling@huanghuai.edu.cn (Y.W.); bailiuyang@huanghuai.edu.cn (L.B.); wangqi@huanghuai.edu.cn (Q.W.); zhangliang@huanghuai.edu.cn (L.Z.); 2 Integrated Nano Optoelectronics Laboratory, University of Michigan, Dearborn, MI 48128, USA; dachuanw@umich.edu (D.W.); yuxiaoz@umich.edu (Y.Z.); xwa@umich.edu (X.W.); 3 Energy Institute, University of Michigan, Ann Arbor, MI 48109, USA

**Keywords:** single nanowires, silicon, dual shells, off-resonance, absorption, photocurrent

## Abstract

Single nanowires (NWs) are of great importance for optoelectronic applications, especially solar cells serving as powering nanoscale devices. However, weak off-resonant absorption can limit its light-harvesting capability. Here, we propose a single NW coated with the graded-index dual shells (DSNW). We demonstrate that, with appropriate thickness and refractive index of the inner shell, the DSNW exhibits significantly enhanced light trapping compared with the bare NW (BNW) and the NW only coated with the outer shell (OSNW) and the inner shell (ISNW), which can be attributed to the optimal off-resonant absorption mode profiles due to the improved coupling between the reemitted light of the transition modes of the leak mode resonances of the Si core and the nanofocusing light from the dual shells with the graded refractive index. We found that the light absorption can be engineered via tuning the thickness and the refractive index of the inner shell, the photocurrent density is significantly enhanced by 134% (56%, 12%) in comparison with that of the BNW (OSNW, ISNW). This work advances our understanding of how to improve off-resonant absorption by applying graded dual-shell design and provides a new choice for designing high-efficiency single NW photovoltaic devices.

## 1. Introduction

Single nanowire (NW) solar cells have attracted more and more research interests as nanoelectronic power sources in recent years due to their unique characteristics, such as enhanced light-harvesting capability, efficient carrier collection, ultra-compact volume, large surface area and convenience of integrating with optoelectronic nanosystems [1,2,3,4,5,6,7,8]. Besides its potential applications as power sources for nanoelectronic devices, single NW solar cell is also helpful to understand the mechanism of the self-assembled NW-based solar cells [7,8,9,10,11,12,13,14,15,16]. It is well known that light-harvesting ability is one of the most critical factors for photovoltaic applications, which determines the photoelectric conversion efficiency of a solar cell. Surprisingly, there is a strong interaction of the incident light with a single NW due to the leaky mode resonances (LMRs), which leads to a much higher absorption cross-section than its physical geometry [17,18,19]. However, the light absorption of single NWs is still far from the expectation due to the sharp resonant peak and narrow width, which can only achieve superior light absorption at the peak position. 

Therefore, various strategies have been implemented to improve the light absorption in the whole solar spectrum range. It was shown that the light absorption could be readily tuned by controlling the size, geometry and orientation of the NWs [20,21,22,23,24,25,26,27,28]. Moreover, our previous studies [29,30,31,32,33] showed that the light absorption could be further improved by introducing a non-absorbing dielectric shell as the antireflection coating, which was experimentally and numerically demonstrated in the recent studies [34,35,36,37,38]. Comparing to the bare NWs (BNWs), semiconductor core-dielectric shell NWs (CSNWs) not only provides the possibility to tune the position of the resonant peak but also enhance the light-harvesting capability at the off-resonant wavelengths by adjusting the thickness and the refractive index of the shell, which is attributed to the high nanofocusing effect [30,38]. However, a further increase of the shell thickness has little contribution to the enhancement of the light absorption of the CSNW structure because the incident light will be mainly concentrated in the dielectric shell. Recently, some new strategies have been employed to improve the light-trapping capability of the CSNW, including the off-axial core-shell design [39] and partially capped design [40]. At the same time, the graded-index concept has been employed to enhance light absorption in the two-dimensional (2D) structures [41,42,43]. However, to the best of our knowledge, the dual shells with the graded refractive index have not been applied to improve the light absorption of single NWs.

In this work, we report a single dual-shell coated NW (DSNW), in which the dual shells have a graded refractive index. We demonstrate that graded dual-shell design can lead to the giant enhancement of off-resonant absorption. The detailed analysis of the absorption mode and photogeneration rate profiles shows that this enhancement results mainly from the optimal off-resonant absorption mode profiles under an improved coupling between the reemitted light of the transition modes of the LMRs of the Si core and the nanofocusing light from the dual shells with the graded refractive index. Simulation results indicate that the photocurrent density is significantly enhanced by 134%, 56% and 12% in comparison with that of the BNW and that of the nanowire only coated with the outer shell (OSNW) and the inner shell (ISNW), respectively.

## 2. Model and Methods 

### 2.1. Model

**Figure 1:**
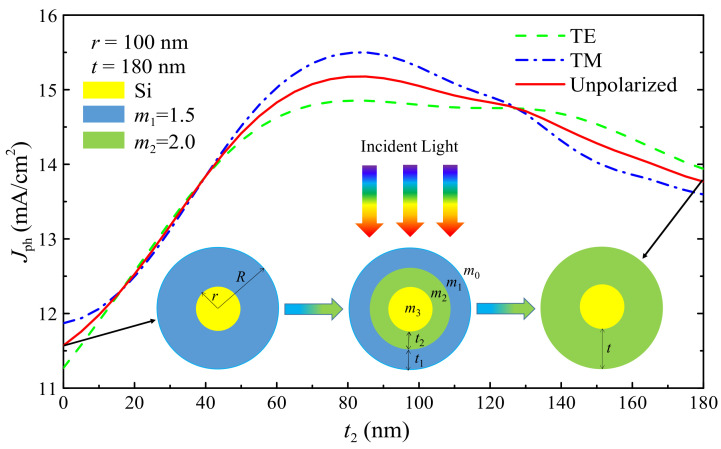
*J*_ph_ versus *t*_2_ of the dual-shell coated nanowire (DSNW) for TE (Transverse electric field to the nanowire axis), TM (Transverse Magnetic field to the nanowire axis) and unpolarized light illumination. The insets are the cross-sectional views of the NW only coated with the outer shell (OSNW) (left), DSNW (middle) and the NW only coated with the outer shell (ISNW) (right), where *J*_ph_ is the photocurrent density; *t*_1_, *t*_2_, *t = t*_1_ + *t*_2_ = 180 nm, *r* = 100 nm and *R = r* + *t =* 280 nm are the outer shell thickness, the inner shell thickness, the total shell thickness, the core radius and the total radius; *m*_3_, *m*_2_, *m*_1_ and *m*_0_ are the complex refractive indices of the Si core, the inner shell, the outer shell and air, respectively.

The cross-sectional schematic diagram of the DSNW is shown in the insets of Figure 1. It should be noted here that the OSNW and the ISNW are also shown for comparison. The geometrical parameters of the DSNW are characterized by the radius *r* (=100 nm) of the Si core, the thickness *t*_1_ of the outer shell and the thickness *t*_2_ of the inner shell of the DSNW which varies from *t*_2_ = 0 nm (i.e., OSNW) to *t*_2_ = 180 nm (i.e., ISNW), the total shell thickness *t* = *t*_1_ + *t*_2_ = 180 nm and the total radius *R* = *r* + *t =* 280 nm. Note here that the radius of the Si core is chosen to be 100 nm as a representative nanoscale size and the total shell thickness is chosen to be 180 nm as the DSNW shows the optimal absorption in this study and the OSNW (or ISNW) approaches the optimal absorption at this thickness in our previous study [31,32,33]. The incident light indicated by colorful arrows in the insets of Figure 1 is assumed to be illuminated perpendicularly to the axial from the top, the wavelength range is from 300 to 1100 nm with a step size of 5 nm considering solar radiation and the bandgap of Si. The wavelength-dependent complex refractive index of Si fitted with the experimental data [44] and that of the inner shell, the outer shell and the surrounding medium (air) are set to be 2.5, 1.5 and 1.0, respectively. Note here that for simplicity, we have neglected the wavelength dependence of the refractive indices to neatly determine the impact of their size on the absorption spectra [30,31,45], as discussed later; however, as long as the wavelength dependence is negligible, our results could apply to any other dielectric with similar refractive index, such as SiO_2_, Si_3_N_4_ and so forth.

### 2.2. Methods

The light absorption performance of the DSNW was performed by solving the corresponding Maxwell’s equations based on 2D finite difference time domain (FDTD) method [46,47,48] by assuming that the length of the NW is far greater than the radius, which can be referred to the work of Kim and co-workers for details [26,27,28,36]. In this simulation, the perfectly matched layers (PML) boundary conditions are used to avoid any non-physical reflection with the boundaries, the total-field scattered-field (TFSF) method was applied to ensure that a single NW interacts with an infinite plane wave. Also, the minimum cell size of the FDTD mesh is set to 1 nm to guarantee the accuracy of the simulation results. 

#### 2.2.1. The Absorption Efficiency and Mode Profile

To qualify the light absorption performance of the DSNW, we define the absorption efficiency *Q*_abs_ of the Si core as [38,49,50,51]:

(1) $$Q_{\mathrm{abs}}=C_{\mathrm{abs}}/C_{\mathrm{geo}},$$

where *C*_geo_ is the geometric cross-section (i.e., the projected area of the Si core) and *C*_abs_ is the absorption cross-section calculated by [38,49,50,51]

(2) $$C_{\mathrm{abs}}=\frac{\int_{V}P_{abs}dV}{I_{0}}=k_{0}{\varepsilon^{\prime \prime }}_{r}\int_{V}{\left|E_{r}\right|}^{2}dV,$$

where *k*_0_ is the wave vector in air, ${\varepsilon^{\prime \prime }}_{r}$ is the imaginary part of the relative permittivity, *E_r_* is the normalized electric field intensity, *V* is the volume of the Si core, *I*_0_ is the solar incident light intensity and *P*_abs_ describes the wavelength-dependent absorption mode profile calculated from Poynting theorem, which can be expressed as [38,49,50,51]

(3) $$P_{abs}=\frac{1}{2}\omega \varepsilon^{\prime \prime }{\left|E\right|}^{2}$$

(4) $$I_{0}=\frac{1}{2}c\varepsilon_{0}{\left|E_{0}\right|}^{2}$$

(5) $${\varepsilon^{\prime \prime }}_{r}=\varepsilon^{\prime \prime }/\varepsilon_{0}=2nk,E_{r}=E/E_{0},$$

where *ω*, *c* and *E*_0_ is the angular frequency, the speed of light and the electric field intensity of the solar incident light; $\varepsilon_{0}$ and $\varepsilon^{\prime \prime }$ are the permittivities in air and the imaginary part of the permittivity of Si; *n* and *k* are the real and imaginary part of the complex refractive index of Si (i.e., *m* = *n* + *ik*, *m*^2^ = $\varepsilon_{r}={\varepsilon^{\prime }}_{r}+i{\varepsilon^{\prime \prime }}_{r}$); *E* is the electric field intensity in the Si core, respectively.

#### 2.2.2. The Photogeneration Rate

With the assumption that each photon absorbed inside the Si core has a contribution to the photocurrent, the spatially dependent photogeneration rate *G* is readily calculated by [52,53]

(6) $$G=\int_{300}^{1100}\frac{P_{abs}}{\hbar \omega }d\lambda =\int_{300}^{1100}\frac{\varepsilon^{\prime \prime }{\left|E\right|}^{2}}{2\hbar }d\lambda ,$$

where *ħ* is the reduced Planck’s constant and *λ* is the wavelength of the incident light.

#### 2.2.3. The Photocurrent Density

To evaluate the light-harvesting capability as single NW solar cells, we can calculate the photocurrent density *J*_ph_ by:

(7) $$J_{\mathrm{ph}}=\frac{q}{C_{geo}}\int_{V}GdV=q\int_{300}^{1100}\Gamma (\lambda )Q_{abs}(\lambda )d\lambda ,$$

where *q* is the element charge, Γ is the AM1.5G standard solar photon flux density spectrum. It should be noted here that 100% collection efficiency is assumed, which has been widely employed to evaluate the ultimate photocurrent [18,53].

#### 2.2.4. The Photocurrent Enhancement Factor (PEF)

To evaluate the photocurrent enhancement of the DSNW, we calculate the photocurrent enhancement factor (PEF) using the relation:

(8) $$\mathrm{PEF}=\left(J_{\mathrm{ph},\mathrm{DSNW}}-J_{\mathrm{ph},\mathrm{rNW}}\right)/J_{\mathrm{ph},\mathrm{rNW}},$$

where *J*_ph,DSNW_ and *J*_ph,rNW_ are the photocurrent density for the DSNW and the reference NWs (BNW, OSNW and ISNW), respectively.

Finally, it is important to stress that the unpolarized illumination (e.g., sunlight) is regarded as the average of transverse electric (TE, electric field normal to the NW axis) and transverse magnetic (TM, magnetic field normal to the NW axis) illumination [22,39,40].

(9) $$Q_{\mathrm{abs}}=\left(Q_{\mathrm{abs}}^{\mathrm{TE}}+Q_{\mathrm{abs}}^{\mathrm{TM}}\right)/2$$

(10) $$J_{\mathrm{ph}}=\left(J_{\mathrm{ph}}^{\mathrm{TE}}+J_{\mathrm{ph}}^{\mathrm{TM}}\right)/2.$$

## 3. Results and Discussion

### 3.1. The Absorption Mechanism

To understand the absorption mechanism responsible for the improved light-harvesting performance of the DSNW, we investigate the photocurrent density (*J*_ph_), the absorption efficiency (*Q*_abs_), the absorption mode profile (*P*_abs_) and the photogeneration rate (*G*), respectively. Note here that *r* = 100 nm, *t* = 180 nm, *R = r + t* = 180 nm, *t*_2_ = 0 → 180 nm, where *t* = 0, *t*_2_ = 0 and *t*_2_ = 180 nm denote the cases of the BNW, OSNW and ISNW, respectively; *m*_3_ is the complex refractive index of the Si core, *m*_2_ = 2.0, *m*_1_ = 1.5 and *m*_0_ = 1.0, as shown in the insets of Figure 1.

#### 3.1.1. The Photocurrent Density (J_ph_)

To evaluate the light-harvesting performance of the DSNW, we first study the effect of the inner shell thickness on the photocurrent density (*J*_ph_) obtained by Equation (7). In Figure 1, we show *t*_2_-dependent *J*_ph_ under normally-incident TE, TM and unpolarized light illumination, respectively. It is observed that *J*_ph_ increases rapidly first when initially increasing *t*_2_, reaches a peak at *t*_2_ = 85 nm and then decreases when continuing to increase *t*_2_. More importantly, *J*_ph_ of the DSNW is always bigger than that of the OSNW as long as the inner shell is adopted and higher than that of the ISNW in a broad inner thickness range of *t*_2_ > 40 nm. For a direct comparison, we list the *J*_ph_ values of the considered BNW, OSNW, ISNW and DSNW configurations under TE, TM and unpolarized illumination, as shown in Table 1. The maximum *J*_ph_ values for TE and TM light are 14.85 and 15.50 mA/cm^2^, respectively. Under unpolarized light illumination (e.g., sunlight), the maximum *J*_ph_ reaches 15.18 mA/cm^2^, which is 96.6%, 31.2% and 10.2% higher than that of the BNW (7.72 mA/cm^2^), OSNW (11.57 mA/cm^2^) and ISNW (13.77 mA/cm^2^), respectively. It is found that this photocurrent enhancement is mainly ascribed to the improvement of *J*_ph_ under any polarized situations (especially TM light), indicating the potential of the DSNW in improving the light absorption of single NWs.

#### 3.1.2. The Absorption Efficiency (Q_abs_)

**Figure 2:**
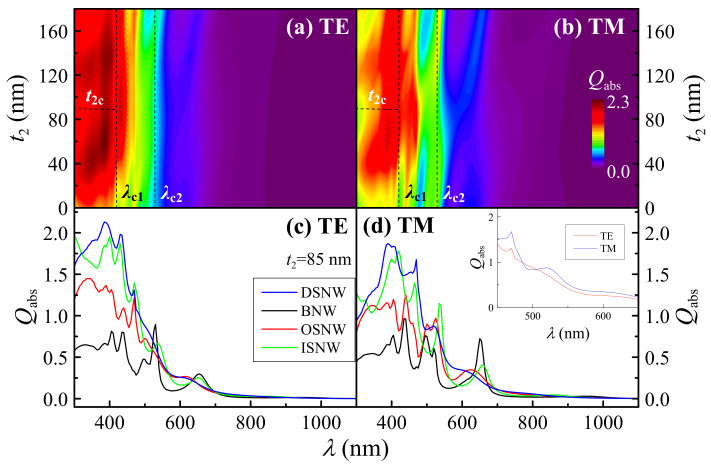
*Q*_abs_ versus *λ* and *t*_2_ of the DSNW for (**a**) TE and (**b**) TM light illumination; *Q*_abs_ versus *λ* of the DSNW (*t*_2_ = 85 nm) under (**c**) TE and (**d**) TM light illumination, together with the BNW (*t* = 0), OSNW (*t*_2_ = 0) and ISNW (*t*_2_ = 180 nm) as references. The inset in (**d**): *Q*_abs_ versus *λ* (450–650 nm) for TE and TM light, where *Q*_abs_ and *λ* are the absorption efficiency and the wavelength of the incident light, respectively. Note that *λ*_c1_ (~430 nm) and *λ*_c2_ (~525 nm) are the characteristic wavelengths denoted by two vertical dashed lines and *t*_2c_ (~90 nm) is the characteristic inner shell thickness in the wavelength range of *λ* < *λ*_c1_ denoted by a horizontal dashed line.

To understand the physical mechanism of the improved photocurrent, we then examine the absorption spectra of the DSNW. In Figure 2a,b, we present 2D maps of *λ*-dependent *Q*_abs_ as a function of *t*_2_ under TE and TM light illumination, which is given by Equation (1). It is clear that the dual-shell design can lead to absorption enhancement in the whole spectrum range compared to the OSNW and almost the entire spectrum range (except several narrow peaks) compared to the ISNW under both TE and TM light illumination (especially at the off-resonant wavelengths), as discussed later. Moreover, *Q*_abs_ can be divided into three regions using two vertical dashed lines by employing the characteristic wavelengths *λ*_c1_ (~430 nm) and *λ*_c2_ (~525 nm) for both TE and TM lights, as labeled in Figure 2a,b. Firstly, in the wavelength range of *λ* < *λ*_c1_, *Q*_abs_ periodically changes with increasing *t*_2_. Note here that *Q*_abs_ can be divided into two regions using a horizontal dashed line by employing the characteristic inner shell thickness *t*_2c_ (~90 nm) for both TE and TM lights, as labeled in Figure 2a,b. *Q*_abs_ reaches the maximum absorption near *t*_2_ = 50 nm and *t*_2_ = 130 nm for the first (*t*_2_ < *t*_2c_) and second (*t*_2_ > *t*_2c_) period, respectively. In other words, the excellent absorption can be obtained in the inner shell thickness range of 40 < *t*_2_ < 60 nm and 110 < *t*_2_ < 140 nm for two periods, respectively. Secondly, in the wavelength range of *λ*_c1_ < *λ* < *λ*_c2_, *Q*_abs_ reaches the maximum absorption near *t*_2_ = 50 nm, that is, the superior absorption can be obtained in the inner shell thickness range of 60 < *t*_2_ < 120 nm. Finally, in the wavelength range of *λ* > *λ*_c2_, *Q*_abs_ appears to be comparable due to the trade-off between the suppression at the resonant wavelengths and the enhancement at the off-resonant wavelengths, resulting in little contribution to the photocurrent enhancement. Therefore, the photocurrent enhancement of the DSNW with *t*_2_ < 60 nm and *t*_2_ > 120 nm is attributed to the improved absorption in the wavelength range of *λ* < *λ*_c1_, while that of the DSNW with 60 < *t*_2_ < 120 nm is mainly attributed to the improved absorption in the wavelength range of *λ*_c1_ < *λ* < *λ*_c2_, which is due to the fact that there is a much higher solar radiation in the wavelength range of *λ*_c1_ < *λ* < *λ*_c2_ than *λ* < *λ*_c1_, leading to a more significant contribution to the photocurrent according to Equation (7).

To quantitatively characterize the absorption enhancement of the DSNW, we also examine the absorption spectra corresponding to the optimal *J*_ph_ in Figure 1. In Figure 2c,d, we show *λ*-dependent *Q*_abs_ of the DSNW with *t*_2_ = 85 nm for TE and TM light, where the results of the BNW, OSNW and ISNW are also included for comparison. It is shown that *Q*_abs_ of the DSNW is much higher than that of the BNW and OSNW in the wavelength range of *λ* < *λ*_c2_ and that of the ISNW in the wavelength range of *λ* < *λ*_c2_ (except for several narrow peaks, for example, *λ* = 470 for TE light) for both TE and TM lights, resulting in a significant photocurrent enhancement. In contrast, although *Q*_abs_ of the DSNW is weaker at the resonant wavelengths, higher at the off-resonant wavelengths than that of all the other three NW structures in the wavelength range of *λ* > *λ*_c2_, leading to a similar contribution to the photocurrent, as discussed above. It is worth noting that *Q*_abs_ can be substantially enhanced at the off-resonant wavelengths over the whole wavelength range for both TE and TM lights, especially for TM light (e.g., near *λ* = 470 nm), which results in the more prominent photocurrent enhancement for TM than TE light. It should also be noted that the match between the absorption efficiency and the solar spectrum becomes another essential factor in evaluating the photocurrent according to Equation (7). For instance, although *Q*_abs_ of the DSNW for TE light is much higher than that for TM light in the wavelength range of *λ* < *λ*_c1_, solar radiation is much lower, which leads to a less photocurrent enhancement, while *Q*_abs_ for TM light is much higher than that for TE light in the wavelength range of 450 < *λ* < 650 nm (except the narrow wavelength range of 490 < *λ* < 505 nm), as shown in the inset of Figure 2d and solar radiation is much higher at the same time, which results in a more significant contribution to the photocurrent.

#### 3.1.3. The Absorption Mode Profile (P_abs_)

**Figure 3:**
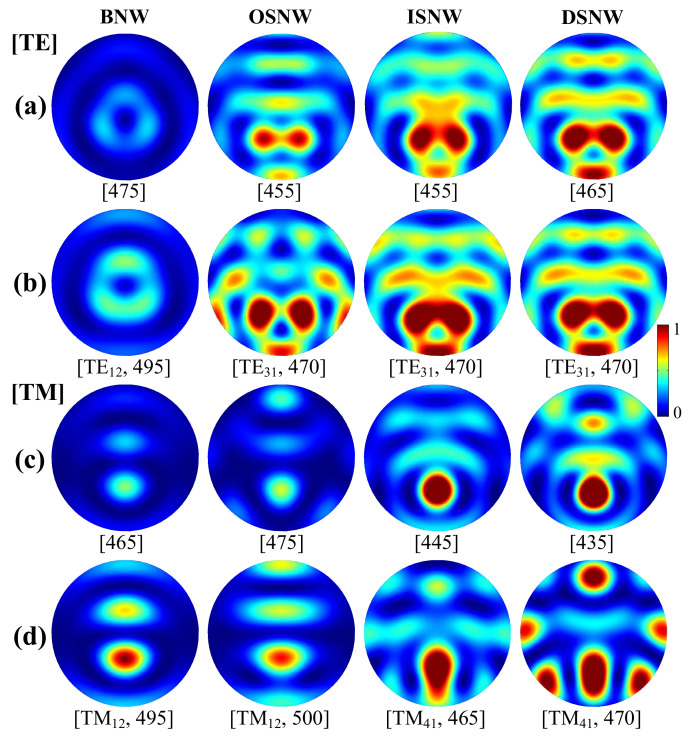
The representative normalized absorption mode profiles inside the Si core corresponding to the wavelengths in Figure 2c,d [from left to right columns are associated with the BNW, OSNW, ISNW and DSNW, respectively]: (**a**,**b**) for TE and (**c**,**d**) for TM light illumination; (**a**,**c**) for off-resonant and (**b**,**d**) for resonant wavelengths. Note that the numbers in square brackets are the wavelengths and the modes of the the leaky mode resonances (LMRs) at the resonant wavelengths are also labeled in the left of square brackets in Figure 3b,d.

The absorption behavior presented above can be well described by the absorption mode profiles (*P*_abs_) calculated by Equation (3) [22,24,26,50,52]. In Figure 3, we examine the normalized absorption mode profiles inside the Si core corresponding to the wavelengths in Figure 2c,d under TE and TM light illumination (these profiles from left to right columns are related to the evolution of the structure from BNW to OSNW and then to ISNW and finally to DSNW). Figure 3a,c show the off-resonant absorption mode profiles for TE and TM light, while Figure 3b,d show the corresponding resonant absorption mode profiles. Note here that the resonant (or off-resonant) absorption for all the four NW configurations may occur at different wavelengths due to the difference of the thickness and the refractive index of the dielectric shells, that is, *t*_1_ (or *t*_2_) or *m*_1_ (or *m*_2_)-driven shift [30,31], as shown in Figure 2a,b. It is observed that the absorption enhancement is attributed to the excitation of the LMRs, likewise in BNW [17,18], which can capture light by multiple total internal reflections at the Si core/inner shell interface when the wavelength of the incident light matches one of the LMRs supported by the Si core. The LMRs can be noted as TE*_ml_* or TM*_ml_*, where *m* and *l* are the azimuthal mode number and the radial order of the resonances, respectively. Figure 3b,d show that the resonant absorption mode profiles of the DSNW are different from that of the BNW, similar to that of the INSW due to the fact that the LMRs of the Si core occur at the Si core/inner shell interface. Specifically, the modes of the BNW, OSNW, ISNW and DSNW are TE_12_, TE_31_, TE_31_ and TE_31_ at *λ* = 495, 470, 470 and 470 nm for TE light and TM_12_, TM_12_, TM_41_ and TM_41_ at *λ* = 495, 500, 465 and 470 nm for TM light, respectively. The absorption of the DSNW is indeed enhanced compared to the BNW and OSNW for both TE and TM lights and slightly suppressed for TE light and enhanced for TM light compared to the ISNW. Figure 3a,c show that the off-resonant absorption mode profiles of the DSNW exhibit a transition mode referred to the LMRs, such transition modes are very close to the corresponding LMRs, which is attributed to the fact that the presence of the graded dual shells makes more light couple into the Si core, leading to a more significant absorption enhancement compared to all the other three NWs.

The absorption behavior presented above can also be well understood by employing the interference effect of light, which occurs at multiple interfaces due to the difference of the refractive index between Si core/inner shell, inner shell/outer shell and outer shell/air. For weaker LMRs in the wavelength of *λ* < *λ*_c2_, as shown in Figure 2, the resonant absorption is greatly enhanced due to the constructive interference with the reemitted light of the weaker LMRs of the Si core. However, for stronger LMRs in the wavelength of *λ* > *λ*_c2_, the resonant absorption is suppressed due to the destructive interference with the reemitted light of the stronger LMRs of the Si core. In contrast, the off-resonant absorption over the whole spectrum is greatly enhanced due to the constructive interference with the reemitted light of the weaker transition modes of the Si core. In a word, the off-resonant (or weaker resonant) absorption is dramatically enhanced owing to an improved coupling between the reemitted light of weaker transition modes (or weaker LMRs) of the Si core and the nanofocusing light from the graded dual shells at the core/inner shell interface [38,39].

#### 3.1.4. The Photogeneration Rate Profile (G)

**Figure 4:**
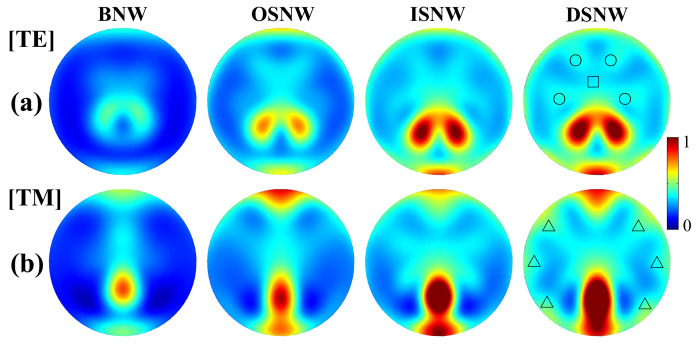
The normalized generation rate profiles of the DSNW for (**a**) TE and (**b**) TM light, together with the BNW, OSNW and ISNW for comparison. Note that the regions labeled by circles and triangles denote that the absorption of the DSNW is significantly enhanced for TE and TM light and the region labeled by the square denotes that the absorption of the DSNW is slightly decreased for TE light, respectively.

To further confirm the physical origin discussed above, we show the photogeneration rate (*G*) obtained Equation (6). In Figure 4, we present the normalized photogeneration rate profiles for TE and TM polarized light, respectively. It is shown in Figure 4a that the absorption of the DSNW for TE light is much stronger than that of the BNW and OSNW and that of almost all the regions of the ISNW [evidently enhanced in the regions labeled by circles (see Figure 4a) and slightly decreased in the region labeled by the square (see Figure 4a)]. It is shown in Figure 4b that the absorption of the DSNW for TM light is also much stronger than that of the BNW and OSNW and that of almost all the regions of the ISNW [evidently enhanced in the regions by labeled by triangles (see Figure 4b)]. These results further reveal that this enhancement arises mainly from the off-resonant absorption enhancement due to the improved coupling between the reemitted light of the weaker transition modes of the LMRs of the Si core and the nanofocusing light from the graded dual shells. More importantly, the photogeneration rate profiles of the DSNW for both TE and TM lights have similar patterns with that of all the other three NWs, again indicating that the absorption enhancement is mainly attributed to the LMRs, likewise in the BNWs.

### 3.2. The Optimization of the Light-Harvesting Performance

**Figure 5:**
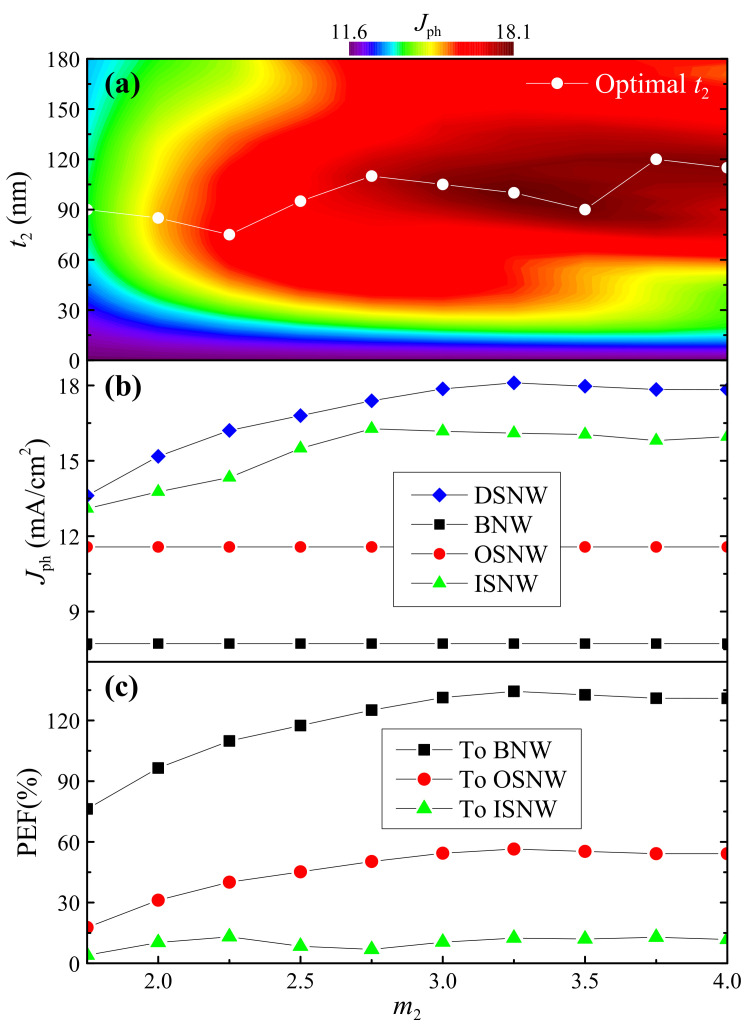
(**a**) *J*_ph_ versus *t*_2_ and *m*_2_ of the DSNW. The white dashed line represents the position of the maximum *J*_ph_ at various *m*_2_ values. (**b**) *J*_ph_ versus *m*_2_ of the DSNW, corresponding to the optimal *t*_2_. Also, *J*_ph_ versus *m*_2_ of the BNW, OSNW and ISNW are included for comparison. (**c**) photocurrent enhancement factor (PEF) versus *m*_2_ of the DSNW compared to the BNW, OSNW and ISNW, respectively. Note that the PEF is the photocurrent enhancement factor compared to the reference NWs.

To evaluate and optimize the light-trapping performance of the DSNW for photovoltaic applications, we now investigate the effect of both the shell thickness and the refractive index on the photocurrent density calculated using Equation (7). Note that except for *m*_2_, all the other structural details of the DSNW are consistent with that shown in the insets of Figure 1. In Figure 4a, we show 2D *J*_ph_ as a function of *t*_2_ and *m*_2_ of the DSNW and the optimal *t*_2_ as a function of *m*_2_. Figure 4a shows *J*_ph_ sharply increases with increasing *t*_2_ at a fixed *m*_2_, reaches its maximum and then decreases when continuing to increase *t*_2_. More importantly, *J*_ph_ of the DSNW is always much larger than that of the OSNW at any *t*_2_ values and higher than that of the ISNW in a broad inner shell thickness range of *t*_2_ > 40 nm for *m*_2_ < 3.5 and *t*_2_ > 60 nm for 3.5 < *m*_2_ < 4.0. It is observed that the maximum values of *J*_ph_ can be obtained in the inner shell thickness range of 90 < *t*_2_ < 110 nm for 3.0 < *m*_2_ < 3.5. In Figure 5b, we show *m*_2_-dependent *J*_ph_ of the DSNW (corresponding to the optimal *t*_2_ in Figure 5a), together with that of the BNW, OSNW and ISNW for comparison. Also, in Figure 5c, we show the photocurrent enhancement factors (PEFs) defined by Equation (8). It is readily observed that *J*_ph_ of the DSNW is much larger than all the other three NWs. In particular, the maximum *J*_ph_ reaches 18.10 mA/cm^2^ at *t*_2_ = 100 nm for *m*_2_ = 3.25, which is 134.4%, 56.4% and 12.4% much larger than that of the BNW (7.72 mA/cm^2^), OSNW (11.57 mA/cm^2^) and ISNW (16.10 mA/cm^2^), respectively.

## 4. Conclusions

In summary, we proposed a single NW by coating dual dielectric shells. The influence of the thickness and the refractive index of the inner shell of the DSNW on the light absorption for photovoltaic applications are numerically investigated. It is found that the size and material of the inner shell can lead to significantly improved off-resonant absorption. The examination of the spatial profiles of the absorption mode and photogeneration rate reveals that the enhancement effect is the result of the constructive interference under the improved coupling between the reemitted light of the transition modes of the LMRs of the Si core and the nanofocusing light from the graded dual shells. The simulation results show that the photocurrent density can be enhanced by 134.4%, 56.4% and 12.4% in comparison with that of the BNW, OSNW and ISNW, respectively. Therefore, such a dual shell coated structure can be applied to a variety of semiconductors to improve the off-resonant absorption and provides an effective way to achieve high-efficiency single NW solar cells.

## Figures and Tables

**Table 1:** Photocurrent densities (in mA/cm^2^) of the four typical configurations (in nm) of the bare NW (BNW), OSNW, ISNW and DSNW under TE (Transverse electric field to the nanowire axis), TM (Transverse Magnetic field to the nanowire axis) and unpolarized light illumination, where *r*, *t*_2_, and *t* are the core radius, the inner shell thickness and the total shell thickness, respectively.

Configuration	Parameter	$J_{\mathrm{ph}}^{\mathrm{TE}}$	$J_{\mathrm{ph}}^{\mathrm{TM}}$	$J_{\mathrm{ph}}$
BNW	*r* = 100, *t*_2_ = 0, *t* = 0	7.24	8.20	7.72
OSNW	*r* = 100, *t*_2_ = 0, *t* = 180	11.27	11.87	11.57
ISNW	*r* = 100, *t*_2_ = 180, *t* = 180	13.94	13.59	13.77
DSNW	*r* = 100, *t*_2_ = 85, *t* = 180	14.85	15.50	15.18

## References

[1] Tian B., Zheng X., Kempa T.J., Fang Y., Yu N., Yu G., Huang J., Lieber C.M. (2007). Coaxial silicon nanowires as solar cells and nanoelectronic power sources. Nature.

[2] Nehra M., Dilbaghi N., Marrazza G., Kaushik A., Abolhassani R., Mishra Y.K., Kim K.H., Kumar S. (2020). 1D semiconductor nanowires for energy conversion, harvesting and storage applications. Nano Energy.

[3] Li Z., Tan H.H., Jagadish C., Fu L. (2018). III–V Semiconductor Single Nanowire Solar Cells: A Review. Adv. Mater. Technol..

[4] Kempa T.J., Day R.W., Kim S.-K., Park H.-G., Lieber C.M. (2013). Semiconductor nanowires: A platform for exploring limits and concepts for nano-enabled solar cells. Energy Environ. Sci..

[5] Christesen J.D., Zhang X., Pinion C.W., Celano T.A., Flynn C.J., Cahoon J.F. (2012). Design principles for photovoltaic devices based on Si nanowires with axial or radial p–n junctions. Nano Lett..

[6] Zhan Y., Li X., Li Y. (2013). Numerical Simulation of Light-Trapping and Photoelectric Conversion in Single Nanowire Silicon Solar Cells. IEEE J. Sel. Top. Quantum Electron..

[7] Tian B., Kempa T.J., Lieber C.M. (2009). Single nanowire photovoltaics. Chem. Soc. Rev..

[8] Tang J., Huo Z., Brittman S., Gao H., Yang P. (2011). Solution-processed core-shell nanowires for efficient photovoltaic cells. Nat. Nanotechnol..

[9] Holm J.V., Jørgensen H.I., Krogstrup P., Nygård J., Liu H., Aagesen M. (2013). Surface-passivated GaAsP single-nanowire solar cells exceeding 10% efficiency grown on silicon. Nat. Commun..

[10] Yuan X., Chen X., Yan X., Wei W., Zhang Y., Zhang X. (2020). Absorption-Enhanced Ultra-Thin Solar Cells Based on Horizontally Aligned p–i–n Nanowire Arrays. Nanomaterials.

[11] Chen W., Roca i Cabarrocas P. (2019). Rational design of nanowire solar cells: From single nanowire to nanowire arrays. Nanotechnology.

[12] Otnes G., Barrigón E., Sundvall C., Svensson K.E., Heurlin M., Siefer G., Samuelson L., Åberg I., Borgström M.T. (2018). Understanding InP Nanowire Array Solar Cell Performance by Nanoprobe-Enabled Single Nanowire Measurements. Nano Lett..

[13] Song K.-D., Kempa T.J., Park H.-G., Kim S.-K. (2014). Laterally assembled nanowires for ultrathin broadband solar absorbers. Opt. Express.

[14] Zhang X., Pinion C.W., Christesen J.D., Flynn C.J., Celano T.A., Cahoon J.F. (2013). Horizontal Silicon Nanowires With Radial p-n Junctions: A Platform for Unconventional Solar Cells. J. Phys. Chem. Lett..

[15] Floris F., Fornasari L., Bellani V., Marini A., Banfi F., Marabelli F., Beltram F., Ercolani D., Battiato S., Sorba L. (2019). Strong Modulations of Optical Reflectance in Tapered Core–Shell Nanowires. Materials.

[16] Floris F., Fornasari L., Marini A., Bellani V., Banfi F., Roddaro S., Ercolani D., Rocci M., Beltram F., Cecchini M. (2017). Self-Assembled InAs Nanowires as Optical Reflectors. Nanomaterials.

[17] Cao L., White J.S., Park J.-S., Schuller J.A., Clemens B.M., Brongersma M.L. (2009). Engineering light absorption in semiconductor nanowire devices. Nat. Mater..

[18] Cao L., Fan P., Vasudev A.P., White J.S., Yu Z., Cai W., Schuller J.A., Fan S., Brongersma M.L. (2010). Semiconductor Nanowire Optical Antenna Solar Absorbers. Nano Lett..

[19] Brönstrup G., Jahr N., Leiterer C., Csáki A., Fritzsche W., Christiansen S. (2010). Optical properties of individual silicon nanowires for photonic devices. ACS Nano.

[20] Kim S., Cahoon J.F. (2019). Geometric Nanophotonics: Light Management in Single Nanowires through Morphology. Acc. Chem. Res..

[21] Choi J.S., Kim K.-H., No Y.-S. (2017). Spatially localized wavelength-selective absorption in morphology-modulated semiconductor nanowires. Opt. Express.

[22] Zhang C., Yang Z., Wu K., Li X. (2016). Design of asymmetric nanovoid resonator for silicon-based single-nanowire solar absorbers. Nano Energy.

[23] Yang Z., Cao G., Shang A., Lei D.Y., Zhang C., Gao P., Ye J., Li X. (2016). Enhanced Photoelectrical Response of Hydrogenated Amorphous Silicon Single-Nanowire Solar Cells by Front-Opening Crescent Design. Nanoscale Res. Lett..

[24] Yang Z., Li X., Lei D.Y., Shang A., Wu S. (2015). Omnidirectional absorption enhancement of symmetry-broken crescent-deformed single-nanowire photovoltaic cells. Nano Energy.

[25] Luo S., Yu W.B., He Y., Ouyang G. (2015). Size-dependent optical absorption modulation of Si/Ge and Ge/Si core/shell nanowires with different cross-sectional geometries. Nanotechnology.

[26] Kim S.-K., Song K.-D., Kempa T.J., Day R.W., Lieber C.M., Park H.-G. (2014). Design of Nanowire Optical Cavities as Efficient Photon Absorbers. ACS Nano.

[27] Kim S.-K., Day R.W., Cahoon J.F., Kempa T.J., Song K.-D., Park H.-G., Lieber C.M. (2012). Tuning Light Absorption in Core/Shell Silicon Nanowire Photovoltaic Devices through Morphological Design. Nano Lett..

[28] Kempa T.J., Cahoon J.F., Kim S.-K., Day R.W., Bell D.C., Park H.-G., Lieber C.M. (2012). Coaxial multishell nanowires with high-quality electronic interfaces and tunable optical cavities for ultrathin photovoltaics. Proc. Natl. Acad. Sci. USA.

[29] Liu W., Oh J.I., Shen W.Z. (2011). Light absorption mechanism in single c-Si (core)/a-Si (shell) coaxial nanowires. Nanotechnology.

[30] Liu W., Oh J.I., Shen W.Z. (2011). Light Trapping in Single Coaxial Nanowires for Photovoltaic Applications. IEEE Electron. Device Lett..

[31] Liu W. (2011). Tunable light absorption of Si nanowires by coating non-absorbing dielectric shells for photovoltaic applications. Optoelectron. Adv. Mater..

[32] Liu W., Hao H. (2015). Enhanced Absorption of Single Silicon Nanowire with Si_3_N_4_ Shell for Photovoltaic Applications. Adv. Mat. Res..

[33] Liu W., Sun F.Z. (2012). Light Absorption Enhancement in Single Si (core)/SiO_2_ (shell) Coaxial Nanowires for Photovoltaic Applications. Adv. Mat. Res..

[34] Zhong Z., Li Z., Gao Q., Li Z., Peng K., Li L., Mokkapati S., Vora K., Wu J., Zhang G. (2016). Efficiency enhancement of axial junction InP single nanowire solar cells by dielectric coating. Nano Energy.

[35] Solanki A., Gentile P., Boutami S., Calvo V., Pauc N. (2015). Dielectric Coating-Induced Absorption Enhancement in Si Nanowire Junctions. Adv. Opt. Mater..

[36] Kim S.-K., Zhang X., Hill D.J., Song K.-D., Park J.-S., Park H.-G., Cahoon J.F. (2015). Doubling Absorption in Nanowire Solar Cells with Dielectric Shell Optical Antennas. Nano Lett..

[37] Yu Y., Ferry V.E., Alivisatos A.P., Cao L. (2012). Dielectric Core–Shell Optical Antennas for Strong Solar Absorption Enhancement. Nano Lett..

[38] Li X., Zhan Y., Wang C. (2015). Broadband enhancement of coaxial heterogeneous gallium arsenide single-nanowire solar cells. Prog. Photovolt. Res. Appl..

[39] Zhang C., Yang Z., Shang A., Wu S., Zhan Y., Li X. (2015). Improved optical absorption of silicon single-nanowire solar cells by off-axial core/shell design. Nano Energy.

[40] Zhou J., Zhang Z., Wu Y., Xia Z., Qin X. (2018). Significantly enhanced coupling to half-space irradiation using a partially capped nanowire for solar cells. Nano Energy.

[41] Saylan S., Milakovich T., Hadi S.A., Nayfeh A., Fitzgerald E.A., Dahlem M.S. (2015). Multilayer antireflection coating design for GaAs_0.69_P_0.31_/Si dual-junction solar cells. Sol. Energy.

[42] Tsai M.-T., Yang Z.-P., Jing T.-S., Hsieh H.-H., Yao Y.-C., Lin T.-Y., Chen Y.-F., Lee Y.-J. (2015). Achieving graded refractive index by use of ZnO nanorods/TiO_2_ layer to enhance omnidirectional photovoltaic performances of InGaP/GaAs/Ge triple-junction solar cells. Sol. Energy Mater. Sol. Cells.

[43] Yeh L.K., Lai K.Y., Lin G.J., Fu P.H., Chang H.C., Lin C.A., He Jr H. (2011). Giant efficiency enhancement of GaAs solar cells with graded antireflection layers based on syringelike ZnO nanorod arrays. Adv. Energy Mater..

[44] Palik E.D. (1985). Handbook of Optical Constants of Solids.

[45] Khudiyev T., Bayindir M. (2014). Superenhancers: Novel opportunities for nanowire optoelectronics. Sci. Rep..

[46] Kane Y. (1966). Numerical Solution of Initial Boundary Value Problems Involving Maxwell’s Equations in Isotropic Media. IEEE Trans. Antennas Propag..

[47] Taflove A., Hagness S.C. (2005). Computational Electrodynamics: The Finite-Difference Time-Domain Method.

[48] Ee H.S., Song K.D., Kim S.K., Park H.G. (2012). Finite-Difference Time-Domain Algorithm for Quantifying Light Absorption in Silicon Nanowires. Isr. J. Chem..

[49] Bohren C.F., Huffman D.R. (1998). Absorption and Scattering of Light by Small Particles.

[50] Zhou L., Yu X., Zhu J. (2014). Metal-Core/Semiconductor-Shell Nanocones for Broadband Solar Absorption Enhancement. Nano Lett..

[51] Mann S.A., Garnett E.C. (2013). Extreme Light Absorption in Thin Semiconductor Films Wrapped around Metal Nanowires. Nano Lett..

[52] Ferry V.E., Polman A., Atwater H.A. (2011). Modeling light trapping in nanostructured solar cells. ACS Nano.

[53] Munday J.N., Atwater H.A. (2010). Large integrated absorption enhancement in plasmonic solar cells by combining metallic gratings and antireflection coatings. Nano Lett..

